# Exploring the most discriminative brain structural abnormalities in ASD with multi-stage progressive feature refinement approach

**DOI:** 10.3389/fpsyt.2024.1463654

**Published:** 2024-10-17

**Authors:** Bingxi Sun, Yingying Xu, Siuching Kat, Anlan Sun, Tingni Yin, Liyang Zhao, Xing Su, Jialu Chen, Hui Wang, Xiaoyun Gong, Qinyi Liu, Gangqiang Han, Shuchen Peng, Xue Li, Jing Liu

**Affiliations:** ^1^ Peking University Sixth Hospital, Peking University Institute of Mental Health, NHC Key Laboratory of Mental Health (Peking University), National Clinical Research Center for Mental Disorders (Peking University Sixth Hospital), Beijing, China; ^2^ Yizhun Medical AI Co., Ltd, Algorithm and Development Department, Beijing, China

**Keywords:** autism spectrum disorder, structural magnetic resonance imaging, feature selection, machine learning, support vector machine, least absolute shrinkage and selection operator

## Abstract

**Objective:**

Autism spectrum disorder (ASD) is a neurodevelopmental condition characterized by increasing prevalence, diverse impairments, and unclear origins and mechanisms. To gain a better grasp of the origins of ASD, it is essential to identify the most distinctive structural brain abnormalities in individuals with ASD.

**Methods:**

A Multi-Stage Progressive Feature Refinement Approach was employed to identify the most pivotal structural magnetic resonance imaging (MRI) features that distinguish individuals with ASD from typically developing (TD) individuals. The study included 175 individuals with ASD and 69 TD individuals, all aged between 7 and 18 years, matched in terms of age and gender. Both cortical and subcortical features were integrated, with a particular focus on hippocampal subfields.

**Results:**

Out of 317 features, 9 had the most significant impact on distinguishing ASD from TD individuals. These structural features, which include a specific hippocampal subfield, are closely related to the brain areas associated with the reward system.

**Conclusion:**

Structural irregularities in the reward system may play a crucial role in the pathophysiology of ASD, and specific hippocampal subfields may also contribute uniquely, warranting further investigation.

## 1 Introduction

Autism spectrum disorder (ASD) is a neurodevelopmental condition characterized by difficulties in social interaction and communication, along with repetitive patterns of behavior, interests, and activities (1). The global prevalence of ASD is currently estimated at around 1%, with an upward trend over time (2). In the United States, the prevalence of ASD among 8-year-old children increased from 1 in 110 in 2006 to 1 in 36 by 2020 (3). In China, a nationwide epidemiological study estimated an ASD prevalence of 0.7% among children aged 6-12 years (4). ASD has a complex etiology and pathogenesis that remain incompletely understood, posing a global public health challenge and imposing a substantial financial burden across various domains, thereby placing pressure on healthcare, social, and political systems of nations (5).

Current research suggests that a combination of genetic and environmental factors leads to abnormal development of certain brain regions, resulting in ASD (6). Consequently, identifying brain regions with structural abnormalities is a fundamental objective in ASD neurobiological research (7). Numerous studies have reported various structural brain abnormalities in individuals with ASD when compared to typically developing (TD) individuals. These anomalies are found in the frontal lobe (8), parietal lobe (9), temporal lobe (10), limbic system (11), and cerebellum (12). They encompass alterations in cortical volume (13), average cortical curvature (14), cortical thickness (15), cortical surface area (16), and other neuroanatomical characteristics. These features may reflect dendritic arborization (17), disrupted intrinsic and extrinsic connectivity patterns (18), or variations in the number of minicolumns within the cortical layer (19). However, these differences are not observed consistently in all studies, possibly due to the heterogeneity among individuals with ASD or methodological differences (20). Given that atypical brain structures underlie functional abnormalities, it is essential to investigate the most distinctive structural brain anomalies associated with ASD. Identifying these distinctive structural brain features in individuals with ASD can advance our understanding of the disorder’s pathogenesis, offer potential biomarkers for more accurate ASD diagnosis, and provide a foundation for exploring more effective interventions.

To identify these distinctive structural brain anomalies in individuals with ASD, the analysis methodology deserves greater attention. Classical statistical methods often overlook the interdependence among different brain regions, which are now recognized as valuable sources of information for detecting various brain disorders (21). In contrast, machine learning approaches prove more suitable for handling extensive and intricate data (22). These methods automatically detect essential patterns among a multitude of features, whether linear or nonlinear, by focusing on specific tasks like classification, thereby emphasizing the significance of meaningful features (23, 24). Furthermore, they enable the assessment of the predictive strength of multiple factors and the identification of the most influential factors contributing to outcomes (25). Consequently, aside from constructing diagnostic models, an increasing number of studies have utilized machine learning to identify prominent features, such as key risk or protective factors for self-harm (26), obesity (27), and bullying (25). In recent years, various machine learning techniques have been employed in ASD classifications. Prior research has validated the effectiveness of these approaches in distinguishing individuals with ASD from those who are typically developing (7, 28). However, contemporary machine learning studies in ASD brain imaging have primarily concentrated on classification performance and early diagnosis, often leading to the generation of an extensive array of features, which poses a challenge in feature reduction (29). Consequently, the ongoing challenge remains the determination of which structural brain deviations are most characteristic of ASD. Furthermore, despite improved accuracy in segmenting subcortical structures, their integration into machine learning classification studies for ASD remains limited. Among subcortical structures, the hippocampus and its subfields have garnered increasing attention in recent years due to their potential relationship with ASD. Researchers suggest that deficits in social behavior, memory, and spatial reasoning in ASD may stem from underlying impairments in complex hippocampal-driven cognitive mechanisms, which are supported by distinct hippocampal subfields (30). Additionally, several animal studies have highlighted the CA2 region of the hippocampus as a crucial structure for social memory (31–33). Additionally, a mouse model of the 22q11.2 deletion syndrome exhibiting ASD-like behaviors has shown age-dependent specific changes in the CA2 area (34). Therefore, exploring changes in hippocampal subfields in individuals with ASD may help identify potential targets for future diagnosis and treatment. However, although the precise segmentation of hippocampal subfields has recently become achievable (35), related research remains exceedingly scarce, and these subfields have scarcely been included in machine learning classification models for ASD.

Therefore, in our present study, we applied a Multi-Stage Progressive Feature Refinement Approach to identify the most crucial structural MRI features that distinguish individuals with ASD from their typically developing counterparts. Within our framework, we integrated cortical features (including thickness, surface area, mean curvature, and volume) as well as subcortical features, particularly focusing on hippocampal subfields. Our research specifically aimed to pinpoint key brain regions that exhibit the most significant distinctions between individuals with autism and typically developing individuals.

## 2 Materials and methods

### 2.1 Participants

Individuals diagnosed with ASD were recruited from Peking University Sixth Hospital in Beijing, China. Inclusion criteria were as follows (1): age between 7 and 18 years; (2) meeting the criteria for ASD according to the Diagnostic and Statistical Manual of Mental Disorders, Fifth Edition (DSM-5); (3) capable of undergoing MRI scanning; (4) right-handed. The diagnosis was confirmed by two experienced child and adolescent psychiatrists based on the DSM-5. Exclusion criteria were as follows: (1) presence of other severe mental disorders such as schizophrenia, bipolar disorder, and so on; (2) major physical or neurological illnesses; (3) history of brain trauma; (4) unstable use of psychotropic medications; (5) presence of metal implants in the body. Typically Developing (TD) participants were recruited through advertisements at Peking University Sixth Hospital. Inclusion criteria for TD participants were: (1) age between 7 and 18 years; (2) absence of mental disorders; (3) capable of undergoing MRI scanning; (4) right-handed. Exclusion criteria for TD groups were: (1) current or previous psychiatric diagnoses; (2) major physical or neurological illnesses; (3) history of brain trauma; (4) use of psychotropic medications; (5) presence of metal implants in the body. Ultimately, our study comprised 175 individuals with ASD and 69 TD participants, matched for gender and age (**Table 1**). In the ASD group, 41 participants had comorbid attention-deficit/hyperactivity disorder (ADHD) and/or Tic disorders, while the rest had no comorbidities. Sixteen participants were on psychotropic medications: four were taking stimulants for ADHD and 12 were on stable doses of antidepressants and/or antipsychotics to manage mood or behavioral issues, with the types and doses of medications remaining stable for at least two weeks prior to the study. The study protocol received approval from the Ethics Committee of Peking University Sixth Hospital. Informed written consent was obtained from parents or legal guardians of participants under 8 years old, and from both participants themselves and their parents or legal guardians for those over 8 years old.

**Table 1:** Demographic information of participants.

Variable	ASD (n=175)	TD (n=69)	*t/χ^2^*	P
	Mean ± SD	Mean ± SD		
Gender (male/female)	154/21	59/10	0.27	0.59
Age	11.93 ± 3.20	12.00 ± 2.91	-0.175	0.86
Full IQ	81.74 ± 26.64(n=173)	110.91 ± 13.21	-7.42	<0.001

### 2.2 Magnetic resonance imaging protocol

MRI scans were conducted using a GE Discovery 750 3.0T MRI system (GE Healthcare, Chicago, IL, USA) equipped with an 8-channel phased-array head coil at both Peking University Sixth Hospital and Peking University Third Hospital. Both scanners were of the same model from the same manufacturer. Subjects were instructed to lie in a supine position and secured with foam padding during the scans. The scanning parameters for the 3D T1-weighted spoiled gradient recalled (SPGR) sequence at Peking University Sixth Hospital were as follows: TR = 6.7 ms, TE = 3.1 ms, flip angle = 12°, FOV = 25.6 mm × 25.6 mm, matrix size = 256 × 256, and slice thickness = 1.0 mm. At Peking University Third Hospital, the scanning parameters for the 3D T1 SPGR sequence were: repetition time (TR) = 4.78 ms, echo time (TE) = 2.02 ms, flip angle = 15°, field of view (FOV) = 24 mm × 24 mm, matrix size = 240 × 240, and slice thickness = 1.0 mm.

### 2.3 Image processing

All T1-weighted images underwent processing with FreeSurfer v.7.2.0(http://surfer.nmr.mgh.harvard.edu/) to extract brain morphometric features. The morphometric procedures of FreeSurfer have consistently demonstrated robust test-retest reliability across different field strengths and scanner manufacturers (36). The processing workflow for cortical reconstruction and subcortical segmentation included several steps: motion correction, skull stripping, computation of the Talairach transform, segmentation of subcortical white matter and deep gray matter volumetric structures, intensity normalization, tessellation of white–gray matter boundaries, automated topology correction, and surface deformation to optimize the placement of structural boundaries (37, 38). We extracted volume, surface area, mean curvature, and thickness of cortical structures defined by Desikan Killiany templates (39), along with the volume of subcortical structures defined by the Aseg template, and the volume of hippocampal subfields using the hippocampal subdivision template. This resulted in a comprehensive set of 317 brain morphometric features for each individual participant. Following the standard processing stream for all images, each image was manually reviewed and corrected to ensure the accuracy of gray/white and gray/pial surface boundaries. In addition to employing several techniques to control head motion during data acquisition, we implemented a FreeSurfer-based quality control workflow for sMRI motion artifacts, which retains only C1 and C2 grade images while discarding C3 grade images (40). To further harmonize MRI data across scanners, we used ComBat, a technique that removes unwanted sources of scan variability while simultaneously enhancing the power and reproducibility of subsequent statistical analyses (41). Specifically, we applied the ComBat method from the sva package in R to correct for batch effects, ensuring more consistent imaging data for our study.

### 2.4 Machine learning analysis

In this study, we employed a Multi-Stage Progressive Feature Refinement Approach that integrates multiple feature selection methods to identify the most discriminative brain structural features for distinguishing ASD from TD. Feature selection in machine learning refers to selecting a subset of features from a larger set that are most strongly correlated with the classification labels and are most important for classification. Common feature selection algorithms include filter methods, embedded methods, and wrapper methods, each with its strengths and weaknesses. Consequently, researchers have increasingly combined these methods for multi-step feature selection (42). In the current study, we combined SelectKBest, the least absolute shrinkage and selection operator (LASSO) algorithm, and support vector machine (SVM). During the selection process, we employed 5-fold cross-validation for internal validation. The data were randomly divided into five parts, using four for training and one for testing, iterating five times. This minimizes the influence of individual anomalous data, providing a comprehensive assessment of model performance across different data subsets. Reducing bias from specific data partitions, it helps prevent overfitting and underfitting. The data were preprocessed for standardization and normalization. All machine learning analyses were implemented in Python programming language using the Scikit-Learn package v. 0.23.2 (http://scikit-learn.org/stable/index.html).

First, we conducted filter feature selection using SelectKBest. SelectKBest utilizes analysis of variance F-values to assess the relationship between features and classification outcomes, selecting the top features with the largest F-values. We used grid search to determine the optimal selection threshold (20%), retaining the top 20% of features ranked by F-score for the next stage. This step ensures the relevance of the selected features to the classification and removes redundancy.

Although the filter selection method considers the relationship between features and classification labels, it does not account for multicollinearity among variables. The LASSO method addresses this by shrinking the coefficients of less important features to zero, reducing the number of features to the most significant ones (43). We used K-fold frequency selection based on LASSO to further refine the features. K-fold frequency selection is an enhanced variant of K-fold cross-validation, focusing on the selection of key features based on their selection frequency. This process generated five feature subsets from the five folds and recorded the frequency of features appearing in these subsets. We retained features selected in more than three out of five folds, ensuring the stability of the selected features and addressing potential redundancy. We used cross-validation to evaluate the effect of different regularization parameters (alpha). Alpha was uniformly sampled on a logarithmic scale from 10^0^ to 10^6^, and the alpha corresponding to the lowest average loss across the 5 folds was selected. The tolerance in the LASSO model was set to 0.0001.

Next, we conducted SVM-based wrapper feature selection to determine the optimal feature set. Wrapper feature selection combines feature selection with the performance of the final classification model to filter the feature set with the highest contribution to classification. SVM has excellent generalization ability in pattern recognition with small samples and high-dimensional data, making it one of the most frequently used classification models in psychiatric brain imaging (44, 45). The kernel for training the SVM model was linear, as this limits the risk of overfitting, contains only a single parameter, and the coefficients of a linear classifier can be interpreted as relative measures of feature importance. The regularization parameter (C) was selected using grid search, and the final value used was C = 10. Classification performance was measured using the following metrics: area under the receiver operating characteristic curve (ROC-AUC), accuracy, sensitivity, and specificity. To ensure robustness and generalization, we used 5-fold cross-validation for internal validation. Features from the previous step were input into the SVM model to derive average contribution rankings using SVM coefficients. We arranged features in descending order based on their rankings and progressively incorporated them into a new SVM model. After adding each feature, we retrained the SVM using four parts of the data and assessed its performance on the excluded part, generating five ROC curves and their respective AUC values, and then calculated the average AUC. As features with decreasing contributions were added, the mean AUC tended to stabilize or converge. Ultimately, we retained the features obtained before the model’s convergence that significantly contributed to performance. These constituted the most discriminative feature set for distinguishing ASD from TD.

## 3 Results

### 3.1 Filter selection based on SelectKBest

In the filter selection based on SelectKBest, we utilized the F-score to identify the top 20% of statistically significant features, resulting in 64 features in each fold. These features include hippocampal subfields such as the volume of the left CA2/3, left CA4, and right subiculum; subcortical structures including the volume of the left nucleus accumbens (NAc), bilateral thalamus, and bilateral pallidum, among others; and various cortical features, such as the surface area of the right caudal anterior cingulate cortex (ACC), the mean curvature of the right dorsolateral prefrontal cortex, and the volume of the left transverse temporal gyrus. These cortical features are distributed across the frontal, temporal, parietal, and occipital lobes.

### 3.2 Frequency selection based on LASSO

**Figure 1:**
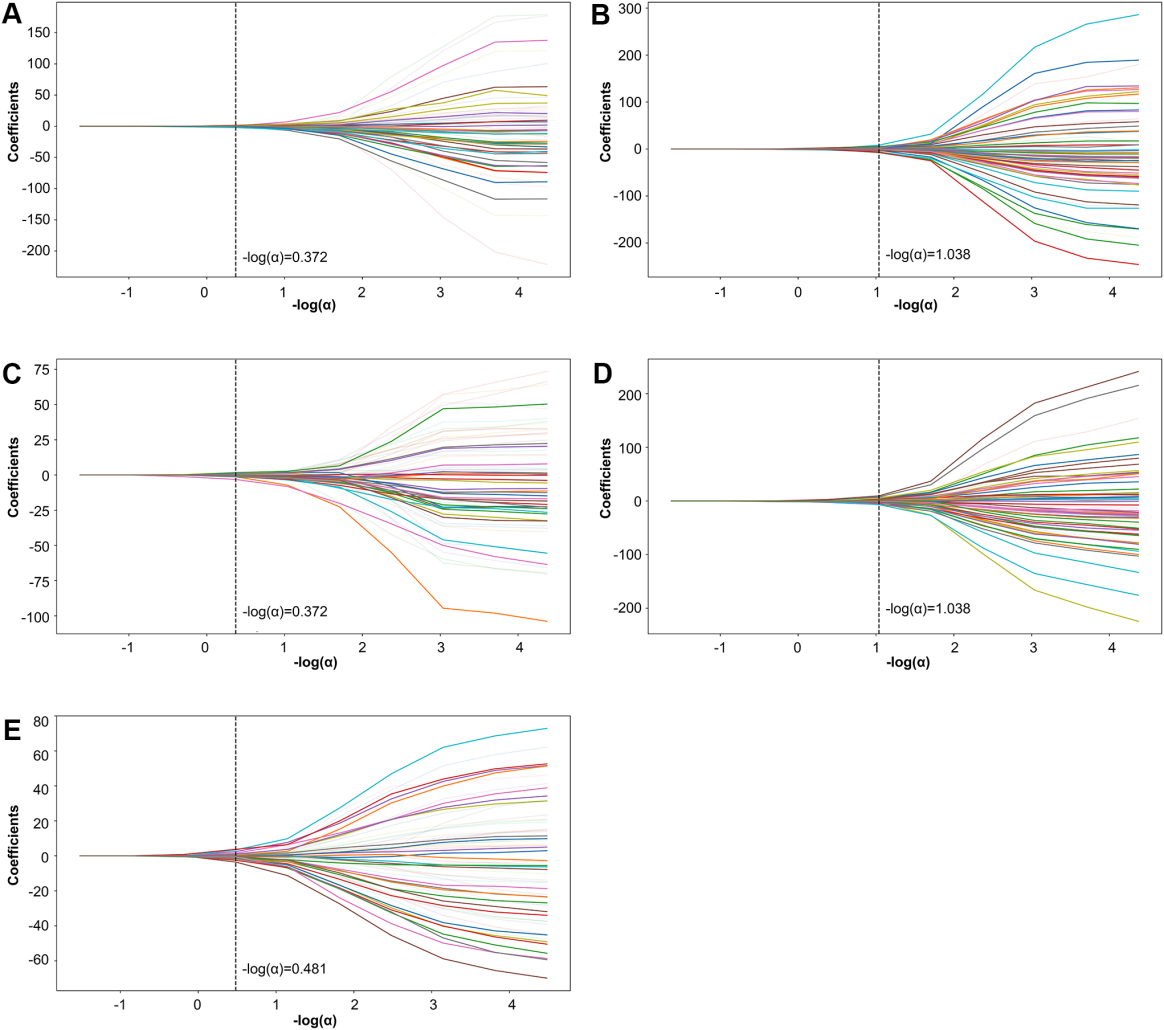
LASSO coefficient path plot. Panels **(A–E)** display the LASSO coefficient paths for each fold. The horizontal axis shows the magnitude of the regularization parameter, while the vertical axis shows the coefficients of the features.

**Figure 2:**
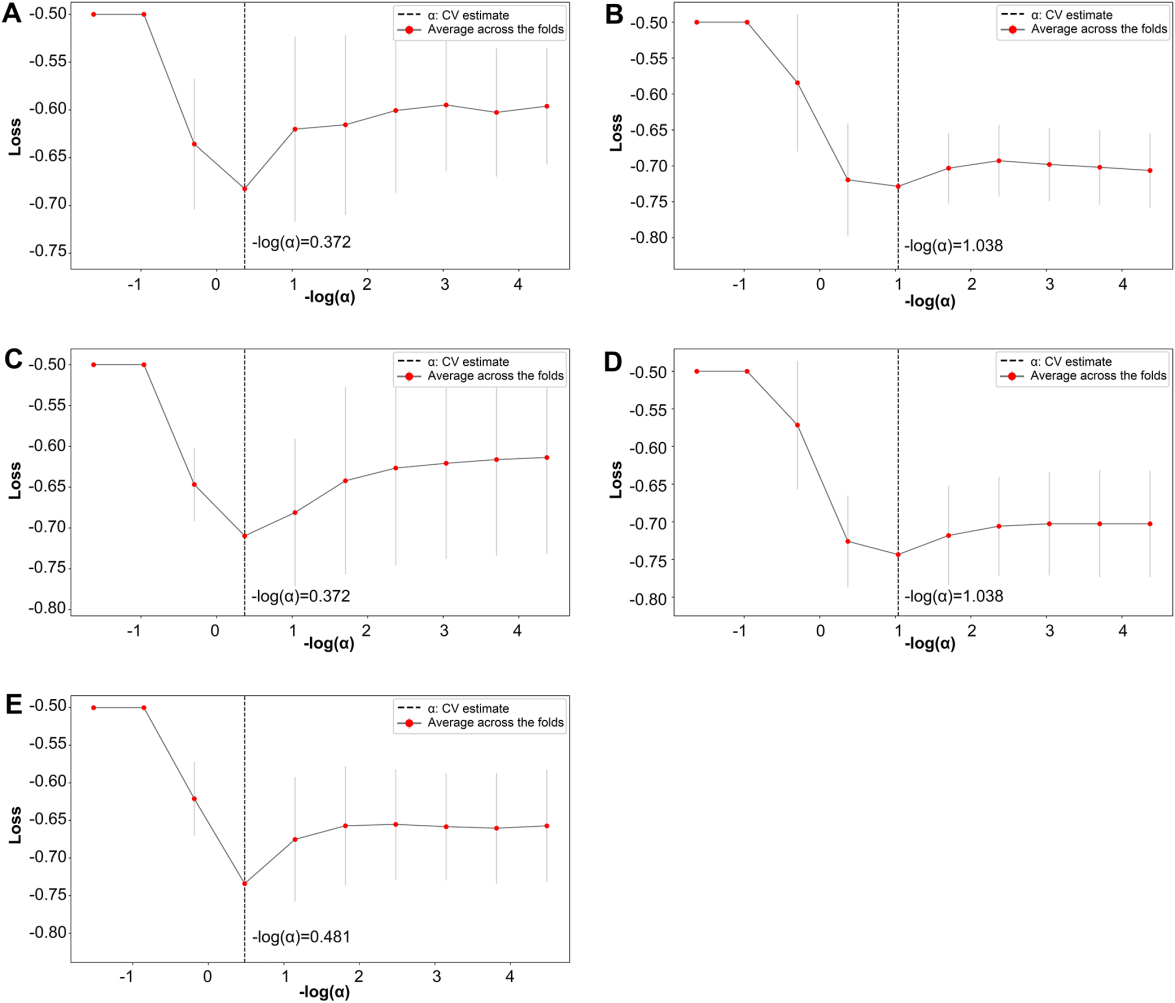
LASSO regularization path plot. In Panels **(A–E)** each curve represents the variation of the LASSO regularization path within a fold. The horizontal axis of the curve represents the magnitude of the regularization parameter, while the vertical axis represents the loss value.

Further refinement using LASSO to reduce redundancy yielded varying numbers of features across the five folds: 31, 47, 28, 53, and 28 features, respectively (**Supplementary Tables S1**-**S5**). This variation indicates the instability of feature selection results due to the partitioning of the training and testing datasets. However, certain features were consistently chosen with high frequency across various folds, demonstrating resilience to dataset partitioning and offering substantial value for identification purposes. A total of 27 features appeared in three or more folds (**Table 2**). Among these, 3 hippocampal subfield features were identified: the volume of left CA2/3, the volume of the right subiculum, and the volume of the left hippocampal tail. The LASSO coefficient path plot (**Figure 1**) and regularization path plot (**Figure 2**) illustrate that in the five-fold cross-validation experiment, the chosen parameters strike a favorable balance between the model’s fitting performance and feature sparsity, indicating that our selection process effectively identifies features that contribute significantly to classification support. There is a significant reduction in the number of features compared to those selected by SelectKBest. However, considerable variation in the coefficients among the features still exists, indicating the potential for further refinement and selection.

**Table 2:** Importance ranking of features based on SVM.

Features	Ranking
left_temporalpole_meancurv	1
right_precuneus_area	2
right_caudalanteriorcingulate_area	3
right_caudalanteriorcingulate_volume	4
left_hipposubfields_CA2/3_volume	5
left_entorhinal_volume	6
left_transversetemporal_volume	7
left_postcentral_thickness	8
left_accumbens_area	9
left_caudalanteriorcingulate_thickness	10
right_pallidum_volume	11
right_cerebellum_cortex_volume	12
left_entorhinal_area	13
right_caudalmiddlefrontal_meancurv	14
right_hipposubfields_subiculum_volume	15
right_thalamus_volume	16
left_hipposubfields_hippocampal_tail_volume	17
right_postcentral_meancurv	18
left_lateralorbitofrontal_meancurv	19
left_parahippocampal_area	20
right_entorhinal_volume	21
right_precentral_meancurv	22
right_parahippocampal_area	23
right_parahippocampal_thickness	24
left_pallidum_volume	25
right_inferiortemporal_thickness	26
estimated_totalintracranial_volume	27

### 3.3 Analysis of the final SVM-based voting selection

**Figure 3:**
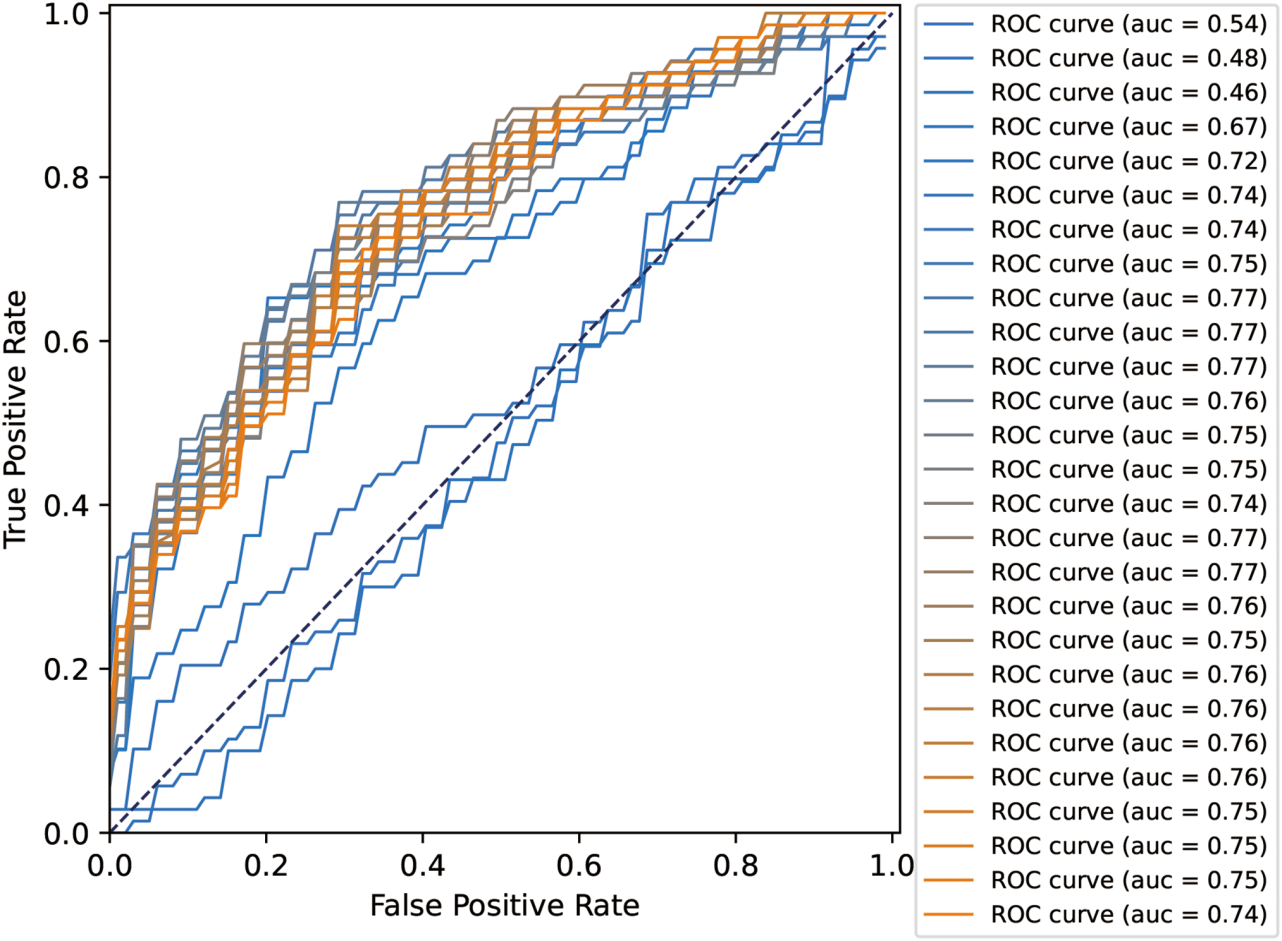
ROC curves and AUC values of the SVM-based voting selection analysis.

All 27 features were initially input into the SVM model, resulting in a ranking of feature importance, as shown in **Table 2**. In the analysis of feature performance based on SVM voting (**Figure 3**), features were incrementally added in descending order of importance to form new feature subsets. These subsets were then inputted into new SVM models. As new features were incorporated, the average AUC value of the test set improved gradually. Furthermore, the contribution of lower-ranked features to classification gradually diminished. The final AUC converged to 0.77 and subsequent addition of low-contributing features had minimal impact on classification improvement. At model convergence, the classification performance was as follows: AUC = 0.77, accuracy = 71.1%, sensitivity = 68.0%, and specificity = 79.7%. This demonstrates the outstanding performance of the selected features. The final set of features that significantly contributed to distinguishing ASD from TD included: mean curvature of the left temporal pole, surface area of the right precuneus, surface area of the right caudal anterior cingulate cortex, volume of the right caudal anterior cingulate cortex, volume of the left CA2/3, volume of the left entorhinal cortex, volume of the left transverse temporal gyrus, cortical thickness of the left postcentral gyrus, and volume of the left nucleus accumbens.

## 4 Discussion

In previous studies, structural abnormalities in various brain regions have been reported among individuals with ASD. However, there has been limited investigation into which specific brain regions exhibit more distinct structural abnormalities in ASD. In our study, we employed a Progressive Feature Refinement Approach to identify nine critical features for distinguishing between individuals with ASD and TD. We integrated both cortical and subcortical features, particularly focusing on hippocampal subfields. These features include: left temporal pole (mean curvature), right precuneus (surface area), right caudal anterior cingulate cortex (surface area), right caudal anterior cingulate cortex (volume), left CA2/3 (volume), left entorhinal cortex (volume), left transverse temporal gyrus (volume), left postcentral gyrus (cortical thickness), left nucleus accumbens (volume). These findings suggest that these specific brain regions may play a more prominent role in the differentiation of individuals with ASD from healthy controls and in the underlying pathophysiology of ASD.

Regarding the subcortical features identified by our method, the regions demonstrating the most robust discriminative power include the left CA2/3 and the left NAc. These subcortical regions have been associated with critical functions in the context of ASD. CA2/3 is one of the hippocampal subfields that has received significant attention in recent years. CA2, in particular, has been consistently identified as essential for social memory in several rodent studies (31–33, 46). Additionally, CA2/3 is closely related to the regulation of oxytocin, which plays an important role in human prosocial behavior. Oxytocin receptors are highly concentrated in CA2/3, and studies have found that these receptors regulate CA2/3’s response to social stimuli (47). These findings suggest that CA2/3 may be involved in the pathophysiology of core symptoms in ASD. However, studies on hippocampal subfields in human subjects with ASD are extremely rare. Li et al. (48, 49) reported that infants with ASD aged 6 to 24 months showed sustained overgrowth in bilateral CA1-3, particularly in the left CA1-3, compared to TD infants. This overgrowth in CA1-3 could serve as a potential biomarker for early ASD diagnosis. In our study, the left CA2/3 volume emerged as a key discriminative feature between ASD and TD, suggesting that CA2/3 abnormalities may persist from infancy through adolescence. This also highlights its potential involvement in the pathophysiology of ASD, warranting further investigation. Unfortunately, in the current FreeSurfer segmentation protocol, the CA2 and CA3 regions cannot be accurately distinguished and are therefore represented as a combined CA2/3 region. Future advancements in more precise segmentation tools may provide valuable insights into the distinct roles of CA2 and CA3 in the pathology of ASD. The NAc is considered a pivotal structure involved in the social reward response (50). Developmental trajectories of the NAc have been observed to differ between individuals with ASD and neurotypical controls (51). Furthermore, a recent meta-analysis of functional magnetic resonance imaging (fMRI) studies found that individuals with ASD exhibit NAc hypoactivation in response to nonsocial rewards, while atypical NAc activation was observed during restricted interests tasks (52). This suggests that NAc dysfunction may be related to the pathological mechanisms underlying ASD. Consequently, NAc is considered a potential target for deep brain stimulation in ASD due to its prominent role in modulating reward and pleasure processing (50). It is evident that these subcortical structures identified by our method hold significant relevance in the pathology of ASD.

Our findings underscore the critical importance of subcortical features in distinguishing individuals with ASD from TD subjects. In previous machine learning studies focused on ASD classification, cortical features have often taken precedence, while subcortical structures have received comparatively less attention. This was primarily due to historical challenges in accurately segmenting subcortical structures and a resulting lack of research emphasis, leading to their relative neglect in subsequent studies (53). However, an increasing body of evidence highlights the relevance of subcortical structures in distinguishing ASD from TD. For instance, Duan et al. (54) mapped the structural covariance network of subcortical regions in groups of young children with ASD and TD. Their findings revealed decreased inter-hemispheric structural covariation, including structural covariance between the left and right thalami, left NAc and right globus pallidus, left globus pallidus and right NAc, and enhanced intra-hemispheric structural covariation in young children with ASD compared to TD individuals. These abnormalities in subcortical structural covariance were predictive of social communication deficits and repetitive and stereotypic behaviors in ASD. Wee et al. (55) found that subcortical volume was significantly superior to other feature types in distinguishing ASD from TD, this advantage is more pronounced in younger subjects (56). Our framework utilized the latest FreeSurfer templates for precise segmentation of subcortical structures and hippocampal subfields. The results suggest that subcortical structural abnormalities may represent a stable feature of ASD pathology and play a significant role in distinguishing individuals with ASD from TD.

It is worth noting that the amygdala, a subcortical structure frequently associated with abnormalities in previous studies (57, 58), was not identified by our framework as one of the most characteristic brain structures in ASD. This observation may be attributed to the age range of our study subjects, which spanned from 7 to 18 years. In individuals with ASD, abnormalities in the amygdala tend to diminish in prominence with age, converging towards sizes found in TD adolescents and adults (59, 60).

In the realm of cortical structures, left temporal pole, right precuneus, right caudal anterior cingulate cortex, left entorhinal cortex, left transverse temporal gyrus, and left postcentral gyrus emerge as the most distinguishing regions. These brain regions have frequently surfaced in previous research on ASD. The caudal anterior cingulate cortex, acting as a processing hub for cognitive control and emotion regulation, integrates both external and internal information, enabling sophisticated control over decisions and behaviors in specific situations (61, 62). Several studies have found structural and functional abnormalities in the right caudal anterior cingulate cortex in individuals with ASD. Ecker et al. (63) observed reductions in the surface area of the anterior cingulate in ASD. Furthermore, the right anterior cingulate cortex has been reported to be significantly smaller in relative volume and less metabolically active in individuals with ASD compared to neurotypical controls (64). The ACC reliably activates when individuals observe emotional faces, listen to emotionally charged voices, or engage in tasks requiring consideration of others’ mental states (65). In ASD males, a significant reduction in self-response in the caudal anterior cingulate cortex was observed during social-exchange games, and this reduction is correlated with the severity of their assessed behavioral symptoms (66). The temporal pole plays crucial roles in socio-emotional processing, advanced semantic processing, theory of mind (ToM), face processing, and autobiographical memory (67, 68). A study investigating cortical characteristics across the lifespan of individuals with ASD found that greater symptom severity in ASD is associated with reduced gray matter volume in the temporal pole (69). Ji et al. (70) discovered that the volume of the temporal pole is associated with the severity of social and speech impairments in patients with ASD who have comorbid developmental delay, with larger volumes linked to greater clinical symptom severity. Rolls et al. (71) reported decreased effective connectivity from the temporal pole to the ventromedial prefrontal cortex in individuals with ASD. Bai et al. (72) found that functional connectivity in the left temporal pole and the left superior temporal gyrus is positively correlated with adaptability and language developmental quotient in children with ASD. The precuneus is crucial for maintaining various important cognitive functions, such as ToM, self-referential processing, episodic memory, and visuospatial processing (73, 74). A meta-analysis found that individuals with ASD have increased gray matter volume in the right precuneus compared to TD subjects (75). Lynch et al. (76) found that in children with ASD, the precuneus demonstrated hypoconnectivity with the visual cortex, basal ganglia, and locally within the posteromedial cortex. A recent study found that the connectivity between the precuneus and temporal lobe is atypically correlated with language expression abilities in infants with ASD, highlighting the precuneus’s involvement in the early neural mechanisms underlying ASD (77). The entorhinal cortex, a key component of the medial temporal lobe and the primary gateway to the hippocampus, is essential for memory and spatial navigation (78). Kwon et al. (79) found that individuals with ASD exhibit reduced gray matter density in the entorhinal cortex compared to controls, highlighting its structural abnormalities in ASD. Additionally, Salmond et al. (80) observed a significant correlation between parental ratings of autistic symptomatology and gray matter density in the entorhinal cortex in individuals with ASD. The transverse temporal gyrus, also known as Heschl’s gyrus, contains the primary auditory cortex and is crucial for auditory processing (81, 82). Heschl’s gyrus is essential not only for processing external auditory stimuli but also for processing ‘inner voices’ (83). Individuals with ASD exhibit various atypical auditory processing patterns, such as hypersensitivity to volume, abnormal pitch perception, and atypical orientation to auditory stimuli, which researchers believe may be related to atypical development of the auditory cortex (84). Reports indicate structural and functional abnormalities of Heschl’s gyrus in individuals with ASD. Hyde et al. (85) demonstrated that adults with autism exhibit increased cortical thickness in Heschl’s gyrus compared to controls. Kim et al. (86)utilized graph theory to examine alterations in different brain network topologies of low-functioning ASD, revealing that increased nodal strength in the right Heschl’s gyrus was positively correlated with the severity of clinical autistic symptoms. The postcentral gyrus, where the primary sensory cortex is located, is known for its central role in processing sensory information from various parts of the body (87). In recent years, sensory sensitivities have been recognized as a core diagnostic feature of ASD, and atypical responses in primary sensory cortices have been observed in autism across sensory modalities and during multimodal perception (88). Mahajan et al. (89) reported that children with ASD have increased gray matter volume in bilateral postcentral gyrus.

A noteworthy characteristic of these highly discriminative brain regions is their substantial overlap with areas associated with the reward system. The reward system supports normal social motivation, facilitating the initiation, maintenance, learning, and adaptation of social interactions (90–92), and consists of the ventral tegmental area, NAc, ACC, orbitofrontal cortex, ventromedial prefrontal cortex, hippocampus, amygdala, thalamus, temporal pole, precuneus (93–95). The “social motivation hypothesis” posits that individuals with ASD experience deficits in processing social rewards, leading to impaired social skills (96). Additionally, multiple fMRI studies have demonstrated that both children and adults with ASD exhibit abnormal responses to both social and monetary rewards (94, 97), indicating that general impairments in reward processing underlie the pathophysiology of autism (94). Such abnormalities in reward processing may contribute to maladaptive motivated behaviors, resulting in a propensity to focus on specific, autism-specific objects and situations rather than conventional environmental rewards (97). Consequently, the social interaction deficits and restricted repetitive behaviors and interests characteristic of ASD may reflect their abnormal functioning of reward circuits in the brain (98). Importantly, critical areas involved in processing social and monetary rewards, such as the NAc, hippocampus, ACC, temporal pole, and precuneus, highly coincide with the regions from which we extracted features. A meta-analysis of previous studies indicated that functional changes in the reward system are associated with the core symptoms of ASD (52). In social reward stimuli tasks, the left hippocampus and anterior cingulate cortex exhibited hypoactivation. In nonsocial reward stimuli tasks, bilateral nucleus accumbens and anterior cingulate cortex showed hypoactivation, while hyperactivation was observed in the right hippocampus. In response to restricted interests, the left nucleus accumbens and anterior cingulate cortex demonstrated hypoactivation, whereas the left precuneus cortex and right nucleus accumbens exhibited hyperactivation. These findings suggest that atypical activation in these reward system structures may contribute to diminished reward responses to social stimuli in individuals with ASD, alongside abnormal reward responses to nonsocial stimuli and restricted interests, potentially leading to social deficits and restricted interests. While previous studies on reward processing in ASD have primarily focused on functional investigations using fMRI, this study provides rare evidence of structural impairments in the reward system of individuals with ASD, indicating that abnormalities in the reward system are not limited to functional changes but also include stable structural alterations in individuals with ASD.

Furthermore, the features with the highest discriminative contribution also show significant overlap with the ToM network. Human social cognitive functions are closely linked with the ToM network, and the theory of mind hypothesis posits that impairments in ToM abilities lead to social impairments in individuals with ASD (99). Dysfunction in the ToM network has been extensively reported in ASD and is confirmed to play a significant role in the disorder’s underlying pathological mechanisms (100–104). The ACC and precuneus are important components of the ToM network and were also included among the most distinctive features identified in our results.

Several limitations of this study should be acknowledged. The first limitation of our study is the age range of our participants, which spanned from 7 to 18 years. Consequently, our findings might not accurately reflect the characteristics of the broader ASD population. Additionally, the ongoing brain development during childhood and adolescence may exhibit complex, age-related patterns of structural changes. The broad age distribution in our study—from childhood to adolescence—may obscure some of these nuanced changes, potentially impacting our results. In future research, we plan to expand the age range and conduct longitudinal studies, aiming to explore the similarities and differences in key brain structural features of ASD across various developmental stages. Secondly, inherent constraints in clinical research led to a limited sample size for the TD group. Future studies should increase the TD sample size to enhance our understanding of atypical brain development in autism. Thirdly, in order to retain a sample that more closely resembles real-world populations, we included participants in the ASD group with IQ below 70, as well as those with comorbid ADHD and/or Tic disorders, and those taking medications. Although excluding these participants entirely could reduce the ecological validity of the study, factors such as IQ, comorbidities, and medication use may still influence the results. Future studies could collect larger samples to further explore these effects. Fourthly, the present study lacked external validation, which is a common limitation of medical studies. Future research should conduct external validation with an independent sample to further verify the model. Fifthly, the SVM model is relatively simple, using straightforward mappings to encode feature relationships. While effective, it may not capture all valuable features. In the future, we can explore more complex interactions between features by introducing advanced deep learning models and interpretable methods based on game theory. Lastly, the selected structural features may indicate developmental abnormalities in ASD or could be the result of functional compensation. Future research is needed to explore these possibilities in greater depth. Despite these limitations, the present study highlights the most crucial structural features for distinguishing between ASD and TD individuals. A strength of our investigation is that we combined different feature selection methods, leveraging their advantages and mitigating their weaknesses, thus effectively removing redundancy. Another advantage is the inclusion of hippocampal subfields. To the best of our knowledge, this is the first study that employs machine learning to investigate the role of hippocampal subfield abnormalities in brain structural anomalies among children and adolescents with ASD. Our findings may help guide future research focused on understanding the role of hippocampal subfields in the pathophysiology of ASD.

In conclusion, our study, employing a Progressive Feature Refinement Approach using LASSO and SVM, identified 9 features out of 317 that significantly distinguish between ASD and TD individuals. These features are closely associated with core impairments in ASD. Notably, both cortical and subcortical structures, including one of the hippocampal subfields, play critical roles and highly overlap with the reward system. Our findings suggest that structural impairments in the brain’s reward system, along with abnormalities in a specific hippocampal subfield, may contribute to a better understanding of the pathophysiology and etiology of ASD. Further research is needed to delve deeper into these findings.

## Data Availability

The datasets presented in this article are not readily available because the data analyzed in this study is subject to the following licenses/restrictions: due to the nature of this research, participants did not consent to their data being shared publicly. Therefore, the supporting data is not available. Requests to access the datasets should be directed to Jing Liu, ljyuch@bjmu.edu.cn.

## References

[1] American Psychiatric Association. Diagnostic and Statistical Manual of Mental Disorders, Fifth Edition. Washington, DC: American Psychiatric Association (2013). doi: 10.1176/appi.books.9780890425596

[2] Zeidan J Fombonne E Scorah J Ibrahim A Durkin MS Saxena S. Global prevalence of autism: A systematic review update. Autism Res. (2022) 15:778–90. doi: 10.1002/aur.2696 PMC9310578 35238171

[3] Maenner MJ Warren Z Williams AR Amoakohene E Bakian AV Bilder DA. Prevalence and characteristics of autism spectrum disorder among children aged 8 years — Autism and developmental disabilities monitoring network, 11 sites, United States, 2020. MMWR Surveill Summ. (2023) 72:1–14. doi: 10.15585/mmwr.ss7202a1 PMC10042614 36952288

[4] Zhou H Xu X Yan W Zou X Wu L Luo X. Prevalence of autism spectrum disorder in China: A nationwide multi-center population-based study among children aged 6 to 12 years. Neurosci Bull. (2020) 36:961–71. doi: 10.1007/s12264-020-00530-6 PMC7475160 32607739

[5] Rogge N Janssen J. The economic costs of autism spectrum disorder: A literature review. J Autism Dev Disord. (2019) 49:2873–900. doi: 10.1007/s10803-019-04014-z 30976961

[6] Squarcina L Nosari G Marin R Castellani U Bellani M Bonivento C. Automatic classification of autism spectrum disorder in children using cortical thickness and support vector machine. Brain Behav. (2021) 11:e2238. doi: 10.1002/brb3.2238 34264004 PMC8413814

[7] Ali MT ElNakieb Y Elnakib A Shalaby A Mahmoud A Ghazal M. The role of structure MRI in diagnosing autism. Diagnostics (Basel). (2022) 12:165. doi: 10.3390/diagnostics12010165 35054330 PMC8774643

[8] Sussman D Leung RC Vogan VM Lee W Trelle S Lin S. The autism puzzle: Diffuse but not pervasive neuroanatomical abnormalities in children with ASD. NeuroImage Clin. (2015) 8:170–9. doi: 10.1016/j.nicl.2015.04.008 PMC4473820 26106541

[9] Wang H Ma Z-H Xu L-Z Yang L Ji Z-Z Tang X-Z. Developmental brain structural atypicalities in autism: a voxel-based morphometry analysis. Child Adolesc Psychiatry Ment Health. (2022) 16:7. doi: 10.1186/s13034-022-00443-4 35101065 PMC8805267

[10] Boddaert N Chabane N Gervais H Good CD Bourgeois M Plumet M-H. Superior temporal sulcus anatomical abnormalities in childhood autism: a voxel-based morphometry MRI study. Neuroimage. (2004) 23:364–9. doi: 10.1016/j.neuroimage.2004.06.016 15325384

[11] Sun F Chen Y Gao Q Zhao Z. Abnormal gray matter structure in children and adolescents with high-functioning autism spectrum disorder. Psychiatry Research: Neuroimaging. (2022) 327:111564. doi: 10.1016/j.pscychresns.2022.111564 36384063

[12] Scott JA Schumann CM Goodlin-Jones BL Amaral DG. A comprehensive volumetric analysis of the cerebellum in children and adolescents with autism spectrum disorder. Autism Res. (2009) 2:246–57. doi: 10.1002/aur.97 PMC2999464 19885834

[13] Schaer M Kochalka J Padmanabhan A Supekar K Menon V. Sex differences in cortical volume and gyrification in autism. Mol Autism. (2015) 6:42. doi: 10.1186/s13229-015-0035-y 26146534 PMC4491212

[14] Hegarty JP Pegoraro LFL Lazzeroni LC Raman MM Hallmayer JF Monterrey JC. Genetic and environmental influences on structural brain measures in twins with autism spectrum disorder. Mol Psychiatry. (2020) 25:2556–66. doi: 10.1038/s41380-018-0330-z PMC6639158 30659287

[15] Ecker C Pretzsch CM Bletsch A Mann C Schaefer T Ambrosino S. Interindividual differences in cortical thickness and their genomic underpinnings in autism spectrum disorder. Am J Psychiatry. (2022) 179:242–54. doi: 10.1176/appi.ajp.2021.20050630 34503340

[16] Boedhoe PSW van Rooij D Hoogman M Twisk JWR Schmaal L Abe Y. Subcortical brain volume, regional cortical thickness, and cortical surface area across disorders: findings from the ENIGMA ADHD, ASD, and OCD working groups. Am J Psychiatry. (2020) 177:834–43. doi: 10.1176/appi.ajp.2020.19030331 PMC8296070 32539527

[17] Huttenlocher PR. Morphometric study of human cerebral cortex development. Neuropsychologia. (1990) 28:517–27. doi: 10.1016/0028-3932(90)90031-I 2203993

[18] Van Essen DC. A tension-based theory of morphogenesis and compact wiring in the central nervous system. Nature. (1997) 385:313–8. doi: 10.1038/385313a0 9002514

[19] McKavanagh R Buckley E Chance SA. Wider minicolumns in autism: a neural basis for altered processing? Brain. (2015) 138:2034–45. doi: 10.1093/brain/awv110 25935724

[20] Katuwal GJ Baum SA Cahill ND Michael AM. Divide and conquer: sub-grouping of ASD improves ASD detection based on brain morphometry. PLoS One. (2016) 11:e0153331. doi: 10.1371/journal.pone.0153331 27065101 PMC4827874

[21] Eslami T Almuqhim F Raiker JS Saeed F. Machine learning methods for diagnosing autism spectrum disorder and attention- deficit/hyperactivity disorder using functional and structural MRI: A survey. Front Neuroinform. (2020) 14:575999. doi: 10.3389/fninf.2020.575999 33551784 PMC7855595

[22] Weller O Sagers L Hanson C Barnes M Snell Q Tass ES. Predicting suicidal thoughts and behavior among adolescents using the risk and protective factor framework: A large-scale machine learning approach. PLoS One. (2021) 16:e0258535. doi: 10.1371/journal.pone.0258535 34731169 PMC8565727

[23] Grossi E Buscema M Della Torre F Swatzyna RJ. The “MS-ROM/IFAST” Model, a novel parallel nonlinear EEG analysis technique, distinguishes ASD subjects from children affected with other neuropsychiatric disorders with high degree of accuracy. Clin EEG Neurosci. (2019) 50:319–31. doi: 10.1177/1550059419861007 31296052

[24] Lu J Kishida K De Asis Cruz J Lohrenz T Deering DT Beauchamp M. Single stimulus fMRI produces a neural individual difference measure for Autism Spectrum Disorder. Clin Psychol Sci. (2015) 3:422–32. doi: 10.1177/2167702614562042 PMC4692192 26722624

[25] Yan W Yuan Y Yang M Zhang P Peng K. Detecting the risk of bullying victimization among adolescents: A large-scale machine learning approach. Comput Hum Behav. (2023) 147:107817. doi: 10.1016/j.chb.2023.107817

[26] Su R John JR Lin P-I. Machine learning-based prediction for self-harm and suicide attempts in adolescents. Psychiatry Res. (2023) 328:115446. doi: 10.1016/j.psychres.2023.115446 37683319

[27] Chatterjee A Gerdes MW Martinez SG. Identification of risk factors associated with obesity and overweight—A machine learning overview. Sensors. (2020) 20:2734. doi: 10.3390/s20092734 32403349 PMC7248873

[28] Nogay HS Adeli H. Machine learning (ML) for the diagnosis of autism spectrum disorder (ASD) using brain imaging. Rev Neurosci. (2020) 31:825–41. doi: 10.1515/revneuro-2020-0043 32866134

[29] Rahman MM Usman OL Muniyandi RC Sahran S Mohamed S Razak RA. A review of machine learning methods of feature selection and classification for autism spectrum disorder. Brain Sci. (2020) 10:949. doi: 10.3390/brainsci10120949 33297436 PMC7762227

[30] Banker SM Gu X Schiller D Foss-Feig JH. Hippocampal contributions to social and cognitive deficits in autism spectrum disorder. Trends Neurosci. (2021) 44:793–807. doi: 10.1016/j.tins.2021.08.005 34521563 PMC8484056

[31] Stevenson EL Caldwell HK. Lesions to the CA2 region of the hippocampus impair social memory in mice. Eur J Neurosci. (2014) 40:3294–301. doi: 10.1111/ejn.12689 PMC4218870 25131412

[32] Hitti FL Siegelbaum SA. The hippocampal CA2 region is essential for social memory. Nature. (2014) 508:88–92. doi: 10.1038/nature13028 24572357 PMC4000264

[33] Smith AS Williams Avram SK Cymerblit-Sabba A Song J Young WS. Targeted activation of the hippocampal CA2 area strongly enhances social memory. Mol Psychiatry. (2016) 21:1137–44. doi: 10.1038/mp.2015.189 PMC4935650 26728562

[34] Piskorowski RA Nasrallah K Diamantopoulou A Mukai J Hassan SI Siegelbaum SA. Age-dependent specific changes in area CA2 of the hippocampus and social memory deficit in a mouse model of the 22q11.2 deletion syndrome. Neuron. (2016) 89:163–76. doi: 10.1016/j.neuron.2015.11.036 PMC4706988 26748091

[35] Weis CN Webb EK Huggins AA Kallenbach M Miskovich TA Fitzgerald JM. Stability of hippocampal subfield volumes after trauma and relationship to development of PTSD symptoms. NeuroImage. (2021) 236:118076. doi: 10.1016/j.neuroimage.2021.118076 33878374 PMC8284190

[36] Han X Jovicich J Salat D van der Kouwe A Quinn B Czanner S. Reliability of MRI-derived measurements of human cerebral cortical thickness: The effects of field strength, scanner upgrade and manufacturer. NeuroImage. (2006) 32:180–94. doi: 10.1016/j.neuroimage.2006.02.051 16651008

[37] Fischl B Salat DH Busa E Albert M Dieterich M Haselgrove C. Whole brain segmentation: automated labeling of neuroanatomical structures in the human brain. Neuron. (2002) 33:341–55. doi: 10.1016/S0896-6273(02)00569-X 11832223

[38] Fischl B Salat DH van der Kouwe AJW Makris N Ségonne F Quinn BT. Sequence-independent segmentation of magnetic resonance images. NeuroImage. (2004) 23:S69–84. doi: 10.1016/j.neuroimage.2004.07.016 15501102

[39] Desikan RS Ségonne F Fischl B Quinn BT Dickerson BC Blacker D. An automated labeling system for subdividing the human cerebral cortex on MRI scans into gyral based regions of interest. Neuroimage. (2006) 31:968–80. doi: 10.1016/j.neuroimage.2006.01.021 16530430

[40] Backhausen LL Herting MM Buse J Roessner V Smolka MN Vetter NC. Quality control of structural MRI images applied using freeSurfer-A hands-on workflow to rate motion artifacts. Front Neurosci. (2016) 10:558. doi: 10.3389/fnins.2016.00558 27999528 PMC5138230

[41] Fortin J-P Cullen N Sheline YI Taylor WD Aselcioglu I Cook PA. Harmonization of cortical thickness measurements across scanners and sites. Neuroimage. (2018) 167:104–20. doi: 10.1016/j.neuroimage.2017.11.024 PMC5845848 29155184

[42] Pudjihartono N Fadason T Kempa-Liehr AW O’Sullivan JM. A review of feature selection methods for machine learning-based disease risk prediction. Front Bioinform. (2022) 2:927312. doi: 10.3389/fbinf.2022.927312 36304293 PMC9580915

[43] Tibshirani R. Regression shrinkage and selection via the lasso. J R Stat Society: Ser B (Methodological). (1996) 58:267–88. doi: 10.1111/j.2517-6161.1996.tb02080.x

[44] Orrù G Pettersson-Yeo W Marquand AF Sartori G Mechelli A. Using Support Vector Machine to identify imaging biomarkers of neurological and psychiatric disease: a critical review. Neurosci Biobehav Rev. (2012) 36:1140–52. doi: 10.1016/j.neubiorev.2012.01.004 22305994

[45] Arbabshirani MR Plis S Sui J Calhoun VD. Single subject prediction of brain disorders in neuroimaging: Promises and pitfalls. Neuroimage. (2017) 145:137–65. doi: 10.1016/j.neuroimage.2016.02.079 PMC5031516 27012503

[46] Miller TD Chong TT-J Aimola Davies AM Johnson MR Irani SR Husain M. Human hippocampal CA3 damage disrupts both recent and remote episodic memories. Elife. (2020) 9:e41836. doi: 10.7554/eLife.41836 31976861 PMC6980860

[47] Raam T McAvoy KM Besnard A Veenema AH Sahay A. Hippocampal oxytocin receptors are necessary for discrimination of social stimuli. Nat Commun. (2017) 8:2001. doi: 10.1038/s41467-017-02173-0 29222469 PMC5722862

[48] Li G Chen M-H Li G Wu D Lian C Sun Q. A longitudinal MRI study of amygdala and hippocampal subfields for infants with risk of autism. Graph Learn Med Imaging (2019). (2019) 11849:164–71. doi: 10.1007/978-3-030-35817-4_20 PMC7043018 32104792

[49] Li G Chen M-H Li G Wu D Lian C Sun Q. Volumetric analysis of amygdala and hippocampal subfields for infants with autism. J Autism Dev Disord. (2023) 53:2475–89. doi: 10.1007/s10803-022-05535-w PMC9537344 35389185

[50] Park HR Kim IH Kang H Lee DS Kim B-N Kim DG. Nucleus accumbens deep brain stimulation for a patient with self-injurious behavior and autism spectrum disorder: functional and structural changes of the brain: report of a case and review of literature. Acta Neurochir (Wien). (2017) 159:137–43. doi: 10.1007/s00701-016-3002-2 27807672

[51] Langen M Schnack HG Nederveen H Bos D Lahuis BE de Jonge MV. Changes in the developmental trajectories of striatum in autism. Biol Psychiatry. (2009) 66:327–33. doi: 10.1016/j.biopsych.2009.03.017 19423078

[52] Clements CC Zoltowski AR Yankowitz LD Yerys BE Schultz RT Herrington JD. Evaluation of the social motivation hypothesis of autism: A systematic review and meta-analysis. JAMA Psychiatry. (2018) 75:797–808. doi: 10.1001/jamapsychiatry.2018.1100 29898209 PMC6143096

[53] Dolz J Desrosiers C Ben Ayed I. 3D fully convolutional networks for subcortical segmentation in MRI: A large-scale study. NeuroImage. (2018) 170:456–70. doi: 10.1016/j.neuroimage.2017.04.039 28450139

[54] Duan X Wang R Xiao J Li Y Huang X Guo X. Subcortical structural covariance in young children with autism spectrum disorder. Prog Neuropsychopharmacol Biol Psychiatry. (2020) 99:109874. doi: 10.1016/j.pnpbp.2020.109874 31981719

[55] Wee C Wang L Shi F Yap P Shen D. Diagnosis of autism spectrum disorders using regional and interregional morphological features. Hum Brain Mapp. (2013) 35:3414–30. doi: 10.1002/hbm.22411 PMC4109659 25050428

[56] Uddin LQ Dajani DR Voorhies W Bednarz H Kana RK. Progress and roadblocks in the search for brain-based biomarkers of autism and attention-deficit/hyperactivity disorder. Transl Psychiatry. (2017) 7:e1218. doi: 10.1038/tp.2017.164 28892073 PMC5611731

[57] Corbett BA Carmean V Ravizza S Wendelken C Henry ML Carter C. A functional and structural study of emotion and face processing in children with autism. Psychiatry Research: Neuroimaging. (2009) 173:196–205. doi: 10.1016/j.pscychresns.2008.08.005 PMC2748131 19665877

[58] Sparks BF Friedman SD Shaw DW Aylward EH Echelard D Artru AA. Brain structural abnormalities in young children with autism spectrum disorder. Neurology. (2002) 59:184–92. doi: 10.1212/wnl.59.2.184 12136055

[59] Chen R Jiao Y Herskovits EH. Structural MRI in autism spectrum disorder. Pediatr Res. (2011) 69:63R–8R. doi: 10.1203/PDR.0b013e318212c2b3 PMC3081653 21289538

[60] Palmen SJMC Durston S Nederveen H Van Engeland H. No evidence for preferential involvement of medial temporal lobe structures in high-functioning autism. Psychol Med. (2006) 36:827–34. doi: 10.1017/S0033291706007215 16512972

[61] Heilbronner SR Hayden BY. Dorsal anterior cingulate cortex: A bottom-up view. Annu Rev Neurosci. (2016) 39:149–70. doi: 10.1146/annurev-neuro-070815-013952 PMC5512175 27090954

[62] Harris LT McClure SM van den Bos W Cohen JD Fiske ST. Regions of the MPFC differentially tuned to social and nonsocial affective evaluation. Cognit Affect Behav Neurosci. (2007) 7:309–16. doi: 10.3758/cabn.7.4.309 18189004

[63] Ecker C Shahidiani A Feng Y Daly E Murphy C D’Almeida V. The effect of age, diagnosis, and their interaction on vertex-based measures of cortical thickness and surface area in autism spectrum disorder. J Neural Transm (Vienna). (2014) 121:1157–70. doi: 10.1007/s00702-014-1207-1 24752753

[64] Haznedar MM Buchsbaum MS Metzger M Solimando A Spiegel-Cohen J Hollander E. Anterior cingulate gyrus volume and glucose metabolism in autistic disorder. Am J Psychiatry. (1997) 154:1047–50. doi: 10.1176/ajp.154.8.1047 9247387

[65] Killgore WDS Yurgelun-Todd DA. Activation of the amygdala and anterior cingulate during nonconscious processing of sad versus happy faces. NeuroImage. (2004) 21:1215–23. doi: 10.1016/j.neuroimage.2003.12.033 15050549

[66] Chiu PH Kayali MA Kishida KT Tomlin D Klinger LG Klinger MR. Self responses along cingulate cortex reveal quantitative neural phenotype for high-functioning autism. Neuron. (2008) 57:463–73. doi: 10.1016/j.neuron.2007.12.020 PMC4512741 18255038

[67] Herlin B Navarro V Dupont S. The temporal pole: From anatomy to function-A literature appraisal. J Chem Neuroanat. (2021) 113:101925. doi: 10.1016/j.jchemneu.2021.101925 33582250

[68] Olson IR Plotzker A Ezzyat Y. The Enigmatic temporal pole: a review of findings on social and emotional processing. Brain. (2007) 130:1718–31. doi: 10.1093/brain/awm052 17392317

[69] Osipowicz K Bosenbark DD Patrick KE. Cortical changes across the autism lifespan. Autism Res. (2015) 8:379–85. doi: 10.1002/aur.1453 25630444

[70] Ji Y Xu M Liu X Dai Y Zhou L Li F. Temporopolar volumes are associated with the severity of social impairment and language development in children with autism spectrum disorder with developmental delay. Front Psychiatry. (2022) 13:1072272. doi: 10.3389/fpsyt.2022.1072272 36532174 PMC9751401

[71] Rolls ET Zhou Y Cheng W Gilson M Deco G Feng J. Effective connectivity in autism. Autism Res. (2020) 13:32–44. doi: 10.1002/aur.2235 31657138

[72] Bai C Wang Y Zhang Y Wang X Chen Z Yu W. Abnormal gray matter volume and functional connectivity patterns in social cognition-related brain regions of young children with autism spectrum disorder. Autism Res. (2023) 16:1124–37. doi: 10.1002/aur.2936 37163546

[73] Cavanna AE Trimble MR. The precuneus: a review of its functional anatomy and behavioural correlates. Brain. (2006) 129:564–83. doi: 10.1093/brain/awl004 16399806

[74] Dadario NB Sughrue ME. The functional role of the precuneus. Brain. (2023) 146:3598–607. doi: 10.1093/brain/awad181 37254740

[75] Nickl-Jockschat T Habel U Michel TM Manning J Laird AR Fox PT. Brain structure anomalies in autism spectrum disorder–a meta-analysis of VBM studies using anatomic likelihood estimation. Hum Brain Mapp. (2012) 33:1470–89. doi: 10.1002/hbm.21299 PMC4801488 21692142

[76] Lynch CJ Uddin LQ Supekar K Khouzam A Phillips J Menon V. Default mode network in childhood autism: posteromedial cortex heterogeneity and relationship with social deficits. Biol Psychiatry. (2013) 74:212–9. doi: 10.1016/j.biopsych.2012.12.013 PMC3710546 23375976

[77] Xiao Y Wen TH Kupis L Eyler LT Taluja V Troxel J. Atypical functional connectivity of temporal cortex with precuneus and visual regions may be an early-age signature of ASD. Mol Autism. (2023) 14:11. doi: 10.1186/s13229-023-00543-8 36899425 PMC10007788

[78] Hafting T Fyhn M Molden S Moser M-B Moser EI. Microstructure of a spatial map in the entorhinal cortex. Nature. (2005) 436:801–6. doi: 10.1038/nature03721 15965463

[79] Kwon H Ow AW Pedatella KE Lotspeich LJ Reiss AL. Voxel-based morphometry elucidates structural neuroanatomy of high-functioning autism and Asperger syndrome. Dev Med Child Neurol. (2004) 46:760–4. doi: 10.1017/s0012162204001306 15540637

[80] Salmond CH Ashburner J Connelly A Friston KJ Gadian DG Vargha-Khadem F. The role of the medial temporal lobe in autistic spectrum disorders. Eur J Neurosci. (2005) 22:764–72. doi: 10.1111/j.1460-9568.2005.04217.x 16101758

[81] Ulanovsky N Las L Nelken I. Processing of low-probability sounds by cortical neurons. Nat Neurosci. (2003) 6:391–8. doi: 10.1038/nn1032 12652303

[82] Henderson D Bichoutar I Moxham B Faidherbe V Plaisant O Guédon A. Descriptive and functional anatomy of the Heschl Gyrus: historical review, manual labelling and current perspectives. Surg Radiol Anat. (2023) 45:337–50. doi: 10.1007/s00276-023-03114-x 36859607

[83] Stephane M Dzemidzic M Yoon G. Keeping the inner voice inside the head, a pilot fMRI study. Brain Behav. (2021) 11:e02042. doi: 10.1002/brb3.2042 33484101 PMC8035434

[84] O’Connor K. Auditory processing in autism spectrum disorder: a review. Neurosci Biobehav Rev. (2012) 36:836–54. doi: 10.1016/j.neubiorev.2011.11.008 22155284

[85] Hyde KL Samson F Evans AC Mottron L. Neuroanatomical differences in brain areas implicated in perceptual and other core features of autism revealed by cortical thickness analysis and voxel-based morphometry. Hum Brain Mapp. (2010) 31:556–66. doi: 10.1002/hbm.20887 PMC6870833 19790171

[86] Kim D Lee JY Jeong BC Ahn J-H Kim JI Lee ES. Overconnectivity of the right Heschl’s and inferior temporal gyrus correlates with symptom severity in preschoolers with autism spectrum disorder. Autism Res. (2021) 14:2314–29. doi: 10.1002/aur.2609 PMC9292809 34529363

[87] Kropf E Syan SK Minuzzi L Frey BN. From anatomy to function: the role of the somatosensory cortex in emotional regulation. Braz J Psychiatry. (2019) 41:261–9. doi: 10.1590/1516-4446-2018-0183 PMC6794131 30540029

[88] Robertson CE Baron-Cohen S. Sensory perception in autism. Nat Rev Neurosci. (2017) 18:671–84. doi: 10.1038/nrn.2017.112 28951611

[89] Mahajan R Dirlikov B Crocetti D Mostofsky SH. Motor circuit anatomy in children with autism spectrum disorder with or without attention deficit hyperactivity disorder. Autism Res. (2016) 9:67–81. doi: 10.1002/aur.1497 25962921 PMC5412258

[90] Rushworth MFS Behrens TEJ Rudebeck PH Walton ME. Contrasting roles for cingulate and orbitofrontal cortex in decisions and social behaviour. Trends Cognit Sci. (2007) 11:168–76. doi: 10.1016/j.tics.2007.01.004 17337237

[91] Grabenhorst F Rolls ET. Value, pleasure and choice in the ventral prefrontal cortex. Trends Cognit Sci. (2011) 15:56–67. doi: 10.1016/j.tics.2010.12.004 21216655

[92] Dichter GS Richey JA Rittenberg AM Sabatino A Bodfish JW. Reward circuitry function in autism during face anticipation and outcomes. J Autism Dev Disord. (2012) 42:147–60. doi: 10.1007/s10803-011-1221-1 PMC8624275 22187105

[93] Dichter GS Rodriguez-Romaguera J. Anhedonia and hyperhedonia in autism and related neurodevelopmental disorders. Curr Top Behav Neurosci. (2022) 58:237–54. doi: 10.1007/7854_2022_312 35397066

[94] Janouschek H Chase HW Sharkey RJ Peterson ZJ Camilleri JA Abel T. The functional neural architecture of dysfunctional reward processing in autism. NeuroImage: Clin. (2021) 31:102700. doi: 10.1016/j.nicl.2021.102700 34161918 PMC8239466

[95] Scott-Van Zeeland AA Dapretto M Ghahremani DG Poldrack RA Bookheimer SY. Reward processing in autism. Autism Res. (2010) 3:53–67. doi: 10.1002/aur.122 20437601 PMC3076289

[96] Chevallier C Kohls G Troiani V Brodkin ES Schultz RT. The social motivation theory of autism. Trends Cognit Sci. (2012) 16:231–9. doi: 10.1016/j.tics.2012.02.007 PMC3329932 22425667

[97] Kohls G Schulte-Rüther M Nehrkorn B Müller K Fink GR Kamp-Becker I. Reward system dysfunction in autism spectrum disorders. Soc Cognit Affect Neurosci. (2013) 8:565–72. doi: 10.1093/scan/nss033 PMC3682440 22419119

[98] Dichter GS Felder JN Green SR Rittenberg AM Sasson NJ Bodfish JW. Reward circuitry function in autism spectrum disorders. Soc Cognit Affect Neurosci. (2012) 7:160–72. doi: 10.1093/scan/nsq095 PMC3277365 21148176

[99] Baron-Cohen S. Theory of mind and autism: A review. Cambridge, MA: Academic Press (2000) p. 169–84. Autism. doi: 10.1016/S0074-7750(00)80010-5

[100] Nijhof AD Bardi L Brass M Wiersema JR. Brain activity for spontaneous and explicit mentalizing in adults with autism spectrum disorder: An fMRI study. NeuroImage Clin. (2018) 18:475–84. doi: 10.1016/j.nicl.2018.02.016 PMC5987841 29876255

[101] Jack A Morris JP. Neocerebellar contributions to social perception in adolescents with autism spectrum disorder. Dev Cognit Neurosci. (2014) 10:77–92. doi: 10.1016/j.dcn.2014.08.001 25170555 PMC6987881

[102] Kana RK Libero LE Hu CP Deshpande HD Colburn JS. Functional brain networks and white matter underlying theory-of-mind in autism. Soc Cognit Affect Neurosci. (2014) 9:98–105. doi: 10.1093/scan/nss106 22977198 PMC3871731

[103] Libero LE Maximo JO Deshpande HD Klinger LG Klinger MR Kana RK. The role of mirroring and mentalizing networks in mediating action intentions in autism. Mol Autism. (2014) 5:50. doi: 10.1186/2040-2392-5-50 25352976 PMC4210608

[104] Cole EJ Barraclough NE Andrews TJ. Reduced connectivity between mentalizing and mirror systems in autism spectrum condition. Neuropsychologia. (2019) 122:88–97. doi: 10.1016/j.neuropsychologia.2018.11.008 30468777

