# Supplementary material for: Exploring the most discriminative brain structural abnormalities in ASD with multi-stage progressive feature refinement approach
Source: Front Psychiatry. 2024 Oct 17;15:1463654. doi: 10.3389/fpsyt.2024.1463654 (PMC11524921; doi:10.3389/fpsyt.2024.1463654)
Supplement: Supplementary file 1 [file Table1.docx]

Supplementary files for “Exploring the Most Discriminative Brain Structural Abnormalities in ASD with Multi-Stage Progressive Feature Refinement Approach”

| **Table S1.** Results of LASSO Screening for Fold 1 | |
| --- | --- |
| **Features** | **Coefficients** |
| left_hipposubfields_CA2/3_volume | 2.380358201 |
| left_accumbens_area | 2.272221953 |
| left_parsopercularis_area | 1.94418835 |
| right_caudalanteriorcingulate_volume | 1.884072488 |
| left_postcentral_thickness | 1.680789321 |
| left_entorhinal_volume | 1.424939319 |
| right_transversetemporal_thickness | 1.36900489 |
| left_parahippocampal_area | 1.152821152 |
| left_superiortemporal_meancurv | 0.94793618 |
| right_inferiorparietal_meancurv | 0.94752692 |
| right_inferiortemporal_thickness | 0.810475375 |
| left_hipposubfields_hippocampal_tail_volume | 0.77759705 |
| left_transversetemporal_volume | 0.769773386 |
| right_pallidum_volume | 0.753543377 |
| left_cerebellum_cortex_volume | 0.751389971 |
| estimated_totalintracranial_volume | 0.650209718 |
| right_medialorbitofrontal_thickness | 0.587635422 |
| right_caudalanteriorcingulate_area | 0.507216257 |
| right_thalamus_volume | 0.486371789 |
| right_caudalmiddlefrontal_meancurv | 0.443957072 |
| left_lateralorbitofrontal_meancurv | 0.376443813 |
| right_parahippocampal_area | 0.338487321 |
| left_pallidum_volume | 0.297471262 |
| right_entorhinal_volume | 0.246096051 |
| right_parstriangularis_meancurv | 0.210242991 |
| right_hipposubfields_subiculum_volume | 0.193625548 |
| right_postcentral_meancurv | 0.158331077 |
| right_precentral_meancurv | 0.134064463 |
| left_lingual_thickness | 0.131562402 |
| left_hipposubfields_CA1_volume | 0.027106311 |
| right_cerebellum_cortex_volume | 0.004394065 |

| **Table S2.** Results of LASSO Screening for Fold 2 | |
| --- | --- |
| **Features** | **Coefficients** |
| right_precuneus_area | 10.74261063 |
| right_postcentral_meancurv | 10.33255675 |
| right_superiorparietal_meancurv | 8.214175217 |
| right_hipposubfields_subiculum_volume | 7.459572369 |
| left_hipposubfields_CA2/3_volume | 7.39674137 |
| right_precuneus_volume | 6.022470466 |
| right_hipposubfields_presubiculum_volume | 4.904983811 |
| right_caudalanteriorcingulate_volume | 4.656542725 |
| left_entorhinal_area | 4.261645178 |
| left_temporalpole_meancurv | 4.206690838 |
| right_precentral_meancurv | 4.129802251 |
| estimated_totalintracranial_volume | 3.90427942 |
| left_accumbens_area | 3.888095254 |
| left_postcentral_thickness | 3.626390096 |
| left_transversetemporal_volume | 3.576021979 |
| right_caudalmiddlefrontal_meancurv | 3.140251871 |
| right_precuneus_meancurv | 3.101265119 |
| right_hipposubfields_CA4_volume | 3.099835698 |
| right_hipposubfields_CA2/3_volume | 3.032971682 |
| left_parahippocampal_area | 2.953109951 |
| right_inferiortemporal_thickness | 2.919126575 |
| left_transversetemporal_area | 2.870808943 |
| right_cerebellum_cortex_volume | 2.580427177 |
| left_hipposubfields_molecular_layer_HP_volume | 2.566478624 |
| right_transversetemporal_volume | 2.532136697 |
| right_supramarginal_meancurv | 2.414781108 |
| right_postcentral_thickness | 2.348327627 |
| right_pallidum_volume | 2.181739807 |
| left_hipposubfields_GC-ML-DG_volume | 1.778081748 |
| left_superiortemporal_volume | 1.521530234 |
| left_hipposubfields_hippocampal_tail_volume | 1.314293318 |
| right_middletemporal_meancurv | 1.070335171 |
| left_lateralorbitofrontal_meancurv | 1.031886114 |
| right_caudalanteriorcingulate_area | 0.99613538 |
| left_cerebellum_whitematter_volume | 0.961988791 |
| rh_hipposubfields_fimbria | 0.81602399 |
| left_pallidum_volume | 0.775099951 |
| left_lingual_thickness | 0.728712007 |
| right_superiorfrontal_area | 0.728066746 |
| left_insula_volume | 0.718503339 |
| left_supramarginal_volume | 0.626014645 |
| left_isthmuscingulate_volume | 0.568596565 |
| right_parahippocampal_thickness | 0.5592992 |
| right_thalamus_volume | 0.278238151 |
| left_medialorbitofrontal_volume | 0.241361729 |
| right_transversetemporal_area | 0.180638781 |
| right_accumbens_area | 0.064336866 |

| **Table S3**. Results of LASSO Screening for Fold 3 | |
| --- | --- |
| **Features** | **Coefficients** |
| left_hipposubfields_CA2/3_volume | 3.376531309 |
| left_entorhinal_volume | 2.172843451 |
| left_bankssts_meancurv | 1.570035882 |
| right_caudalanteriorcingulate_volume | 1.55582024 |
| right_precuneus_area | 1.532185185 |
| left_postcentral_thickness | 1.488991981 |
| left_posteriorcingulate_area | 1.431370739 |
| right_caudalanteriorcingulate_area | 1.419730361 |
| left_temporalpole_meancurv | 1.415265585 |
| right_rostralanteriorcingulate_thickness | 1.244294604 |
| left_caudalanteriorcingulate_thickness | 1.112084467 |
| left_thalamus_volume | 1.101253021 |
| right_pallidum_volume | 1.060665248 |
| left_posteriorcingulate_meancurv | 0.799881783 |
| left_entorhinal_area | 0.758570374 |
| right_cerebellum_whitematter_volume | 0.718710779 |
| right_isthmuscingulate_volume | 0.712837654 |
| right_entorhinal_volume | 0.70719624 |
| right_hipposubfields_subiculum_volume | 0.627889748 |
| left_isthmuscingulate_volume | 0.592823032 |
| right_thalamus_volume | 0.496712007 |
| left_pallidum_volume | 0.416708288 |
| left_accumbens_area | 0.376262713 |
| left_transversetemporal_volume | 0.317956805 |
| left_insula_volume | 0.2720136 |
| left_parahippocampal_area | 0.239641344 |
| right_cuneus_volume | 0.062158542 |
| left_caudate_volume | 0.046579017 |

| **Table S4**. Results of LASSO Screening for Fold 4 | |
| --- | --- |
| **Features** | **Coefficients** |
| left_parsopercularis_area | 10.30681223 |
| right_postcentral_meancurv | 8.634827324 |
| left_hipposubfields_molecular_layer_HP_volume | 7.746780077 |
| right_hipposubfields_subiculum_volume | 7.543311732 |
| left_postcentral_meancurv | 7.343512607 |
| left_hipposubfields_CA2/3_volume | 6.708783715 |
| left_postcentral_thickness | 6.395157805 |
| left_inferiorparietal_meancurv | 5.910476544 |
| right_precentral_thickness | 5.889101997 |
| right_precuneus_area | 5.627708977 |
| right_precentral_meancurv | 4.947685039 |
| right_inferiorparietal_meancurv | 4.928589988 |
| right_parahippocampal_area | 4.410665906 |
| left_frontalpole_thickness | 4.326144826 |
| left_superiortemporal_meancurv | 4.188053065 |
| left_lateraloccipital_meancurv | 3.698170778 |
| left_temporalpole_meancurv | 3.581535624 |
| right_cuneus_volume | 3.296468957 |
| right_cerebellum_cortex_volume | 3.285338734 |
| right_caudalanteriorcingulate_volume | 3.245953613 |
| right_lateraloccipital_meancurv | 2.998986236 |
| left_parsopercularis_volume | 2.889278193 |
| left_caudalanteriorcingulate_thickness | 2.535730115 |
| right_precuneus_meancurv | 2.430514566 |
| right_inferiortemporal_thickness | 2.3429877 |
| right_hipposubfields_CA4_volume | 2.328448275 |
| left_bankssts_meancurv | 2.244880706 |
| right_caudalmiddlefrontal_meancurv | 2.13470216 |
| right_bankssts_meancurv | 1.910167128 |
| right_isthmuscingulate_area | 1.897652059 |
| right_isthmuscingulate_volume | 1.828277762 |
| left_accumbens_area | 1.795102978 |
| right_inferiortemporal_meancurv | 1.75155642 |
| left_hipposubfields_hippocampal_tail_volume | 1.64579552 |
| left_precentral_meancurv | 1.632545752 |
| right_hipposubfields_GC-ML-DG_volume | 1.340207752 |
| right_superiorparietal_meancurv | 1.28723611 |
| left_pallidum_volume | 1.262828985 |
| left_superiorfrontal_meancurv | 1.228609819 |
| right_supramarginal_meancurv | 1.188872095 |
| right_caudalanteriorcingulate_area | 1.12643742 |
| left_middletemporal_meancurv | 0.977675424 |
| left_lateralorbitofrontal_meancurv | 0.921897002 |
| left_entorhinal_volume | 0.918651051 |
| right_hipposubfields_CA2/3_volume | 0.86746606 |
| right_parsorbitalis_thickness | 0.806374738 |
| right_lateralorbitofrontal_thickness | 0.759508433 |
| left_parahippocampal_area | 0.727601886 |
| right_parahippocampal_thickness | 0.693472569 |
| estimated_totalintracranial_volume | 0.662866601 |
| left_posteriorcingulate_meancurv | 0.474282858 |
| left_parahippocampal_thickness | 0.244847716 |
| right_middletemporal_meancurv | 0.243191484 |

| **Table S5**. Results of LASSO Screening for Fold 5 | |
| --- | --- |
| **Features** | Coefficients |
| left_precuneus_area | 4.606760059 |
| right_caudalanteriorcingulate_volume | 4.355296758 |
| right_temporalpole_area | 3.845039863 |
| left_caudalanteriorcingulate_thickness | 3.093214875 |
| left_entorhinal_area | 2.983086758 |
| right_caudalanteriorcingulate_thickness | 2.29270846 |
| left_temporalpole_meancurv | 2.079766204 |
| left_transversetemporal_volume | 1.767706695 |
| left_lateraloccipital_meancurv | 1.7245876 |
| right_hipposubfields_subiculum_volume | 1.613018119 |
| right_lateralorbitofrontal_thickness | 1.45045669 |
| right_pallidum_volume | 1.412540169 |
| right_caudalmiddlefrontal_meancurv | 1.400321072 |
| right_entorhinal_volume | 1.243593623 |
| left_accumbens_area | 1.109880069 |
| right_parahippocampal_area | 0.89870737 |
| left_frontalpole_thickness | 0.828315257 |
| right_precentral_thickness | 0.66809484 |
| left_rostralanteriorcingulate_area | 0.656477214 |
| left_caudate_volume | 0.656137773 |
| left_hipposubfields_CA2/3_volume | 0.556937445 |
| left_superiorparietal_area | 0.483958856 |
| left_hipposubfields_CA4_volume | 0.427958602 |
| left_parahippocampal_thickness | 0.383887091 |
| left_pallidum_volume | 0.371288733 |
| right_inferiortemporal_thickness | 0.248171403 |
| left_caudalanteriorcingulate_volume | 0.198210683 |
| right_parahippocampal_thickness | 0.153841158 |

*Note*: Tables S1~S5 display the features selected by LASSO in the 5-fold cross-validation, along with the corresponding coefficients. The coefficients represent the weights of the features in the LASSO model.
